# Intragenomic Long-Distance RNA–RNA Interactions in Plus-Strand RNA Plant Viruses

**DOI:** 10.3389/fmicb.2018.00529

**Published:** 2018-04-04

**Authors:** Tamari Chkuaseli, K. Andrew White

**Affiliations:** Department of Biology, York University, Toronto, ON, Canada

**Keywords:** plant virus, RNA virus, RNA structure, RNA regulation, RNA–RNA interaction, translation, virus replication, tombusvirus

## Abstract

Plant viruses that contain positive-strand RNA genomes represent an important class of pathogen. The genomes of these viruses harbor RNA sequences and higher-order RNA structures that are essential for the regulation of viral processes during infections. In recent years, it has become increasingly evident that, in addition to locally positioned RNA structures, long-distance intragenomic interactions, involving nucleotide base pairing over large distances, also contribute significantly to the control of various viral events. Viral processes that are modulated by such interactions include genome replication, translation initiation, translational recoding, and subgenomic mRNA transcription. Here, we review the structure and function of different types of long-distance RNA–RNA interactions, herein termed LDRIs, present in members of the family *Tombusviridae* and other plus-strand RNA plant viruses.

## Introduction

Plus-strand RNA viruses comprise the largest group of plant viruses, many of which are significant pathogens. During infections, the RNA genomes of these viruses serve multiple functions, including acting as messages for translation of viral proteins, templates for viral RNA replication and transcription, and genetic cargo for packaging. Collectively, these viruses use a wide variety of strategies to control these types of processes, and RNA elements within their genomes play integral roles in such regulation (Newburn and White, 2015).

Traditionally, functional viral RNA elements composed of higher-order structures have been viewed as entities that are relatively localized, such as promoters positioned at genomic termini. However, there is now mounting evidence that a more holistic view of RNA genomes is required to fully appreciate the full diversity of RNA-based regulation (Nicholson and White, 2015). For example, many plant viruses harbor RNA elements located internally within their genomes, some of which reside in coding regions (Newburn and White, 2015). Even more astonishing is the growing list of plus-strand RNA viruses that utilize intragenomic long-distance RNA–RNA interactions (LDRIs) as part of their regulatory system (Miller and White, 2006; Nicholson and White, 2014). Notably, this inventory includes a significant number of plant viruses, many of which belong to the family *Tombusviridae* (Russo et al., 1994). Within this family, the genus *Tombusvirus*, typified by *Tomato bushy stunt virus* (TBSV), represents, to date, the most extreme example of LDRI utilization (White and Nagy, 2004). Similarly, other genera in this large family also employ LDRIs to varying degrees, establishing members of *Tombusviridae*, termed tombusvirids, as a discrete group that routinely employs this non-conventional form of RNA-based regulation. The LDRI strategy, however, is not limited to tombusvirids, because other examples have been identified in diverse genera, including *Luteoviruses, Nepoviruses*, and *Potexviruses* (Newburn and White, 2015).

Long-distance RNA–RNA interactions can span significant distances within viral RNA genomes, and the sequences traversed can range from about one thousand to several thousand nucleotides (Miller and White, 2006; Nicholson and White, 2014; Newburn and White, 2015). In some cases, the intervening sequence is predicted to form a distinct RNA secondary structure domain that could assist in bringing complementary sequences together (Wu et al., 2013). Nucleotide composition of the interacting sequences generally vary and are usually evenly distributed between CG and AU pairs. The partner segments forming the interactions can be as short as 5 nts in length or as long as 11 nts. Bulges or mismatches within the regions of complementarity are rare; however, GU wobble pairs are present in some interactions. Although standard Watson–Crick base pairing is central to these interactions, it is also possible that non-canonical or stacking interactions, as well as protein binding, could further stabilize these contacts. However, because these interactions are dynamic, a balance between stability and liability is required.

The local structural contexts of partner sequences are often, but not always, within terminal loops, internal loops, or bulges, which facilitate their presentation for base pairing (Simon and Miller, 2013; Nicholson and White, 2014; Newburn and White, 2015). Within the viral genome, many of these sequences reside in coding regions. Accordingly, the LDRI function of an RNA must be integrated with its coding function, leading to a compromise that adequately satisfies both activities. Similarly, the operation of LDRIs must also be coordinated with other potentially interfering viral processes, such as translation and replication of the viral RNA. In cases where multiple LDRIs exist in a single genome, further regulation must exist to determine when each interaction occurs and for what length of time.

The study of LDRIs is a growing field that is constantly uncovering new examples of this atypical form of RNA-based regulation. Here, we provide a current overview of the involvement of LDRIs in the reproductive cycles of various plus-strand RNA plant viruses.

## Tombusvirus: a Paradigm for LDRIs

**FIGURE 1:**
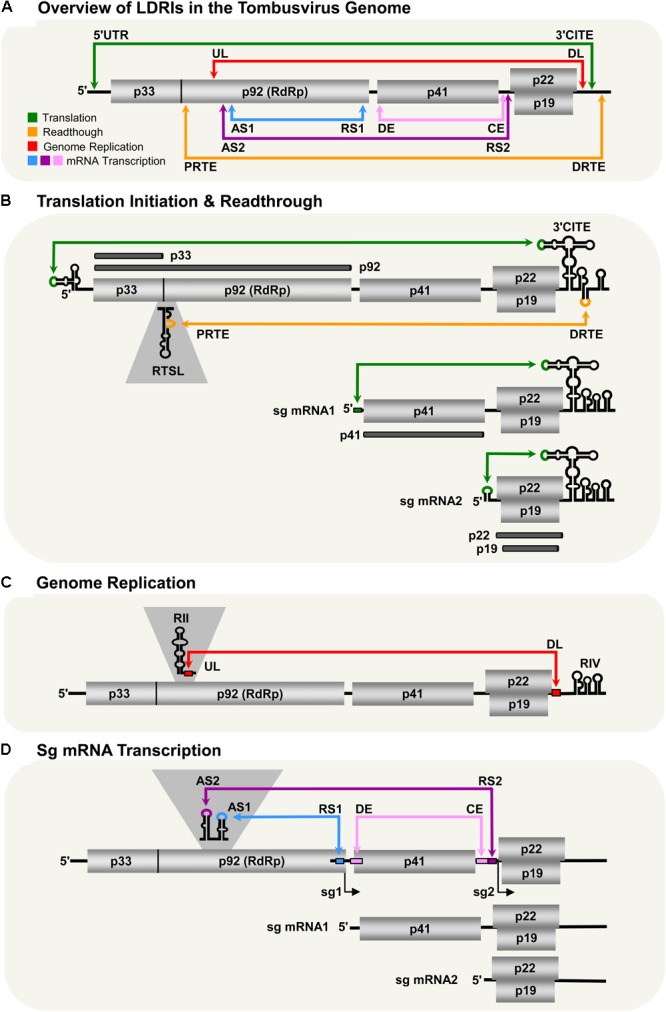
LDRIs in *Tomato bushy stunt virus* (TBSV) genomic and sg mRNAs. Genomic and sg mRNAs of TBSV are represented linearly with the ORFs encoding proteins shown as gray boxes. **(A)** Overview of LDRIs in the TBSV genome. The six different LDRIs currently known are color-coded and represented by double-headed arrows that point to the relative positions of partner sequences along with their identifying acronyms. Genome coordinates of LDRI sequences: 5′-UTR (21–30), 3′-CITE (4522–4531), PRTE (1134–1140), DRTE (4739–4744), UL (1455–1465), DL (4410–4420), AS1 (1607–1613), RS1 (2612–2618), AS2 (1567–1571), RS2 (3832–3838), DE (2639–2674), CE (3804–3830). **(B)** Translation initiation and readthrough. The TBSV genome and its two sg mRNAs are shown. RNA structures relevant to these processes are depicted in the 5′-UTRs, readthrough region (RTSL, readthrough stem–loop) and 3′-UTRs (3′-CITE). The green arrows denote LDRIs required for translation initiation (occurring between the 5′-UTR and 3′-CITE) and the orange arrow represents the LDRI required for readthrough (between the PRTE and DRTE). The thick dark horizontal lines represent the proteins translated from the respective viral RNAs. **(C)** Genome replication. RNA structures relevant to replication (RII and RIV) are shown along with the UL and DL sequences that form the required LDRI (red). **(D)** Sg mRNA transcription. The LDRIs required to form the AS–RS attenuation structures for transcription of sg mRNA1 (blue) or sg mRNA2 (purple) are shown, along with the auxiliary LDRI formed by DE and CE (pink) required for sg mRNA2.

*Tombusvirus* is the prototype genus in the family *Tombusviridae* (White and Nagy, 2004). These viruses are extraordinary because they require the formation of at least six distinct intragenomic LDRIs during infections. The genus is typified by TBSV, which possesses an icosahedral capsid containing a single-stranded, monopartite, messenger-sense RNA genome of ∼4.8 kb (**Figure 1A**; Hearne et al., 1990). The genome encodes five functional proteins flanked by 5′- and 3′-untranslated regions (UTRs) of 166 and 352 nt, respectively. Positioned 5′-proximally is the open-reading frame (ORF) for an auxiliary replication protein, p33. Translational readthrough of the p33 ORF results in the production of the p92 RNA-dependent RNA-polymerase (RdRp) (Gunawardene et al., 2017). Encoded further downstream is the capsid protein (p41), and then overlapping ORFs for the cell-to-cell movement (p22) and suppressor of antiviral RNA silencing (p19) proteins. These proteins are translated from two subgenomic (sg) mRNAs that are transcribed from the viral genome. During the course of an infection, TBSV proteins are expressed in defined amounts at specific times and, in all cases, LDRIs are involved in mediating the steps in their production. Moreover, LDRIs are also involved in regulating replication of the viral RNA genome.

### Translation Initiation

Tombusvirus RNA genomes lack both a 5′-cap structure and a 3′-poly(A) tail, which are typical terminal modifications required for efficient initiation of translation (White and Nagy, 2004). These viruses overcome this deficiency by employing an RNA structure positioned in the 3′-UTRs of their genomes that acts as a cap-independent translational enhancer or 3′-CITE (Wu and White, 1999; Fabian and White, 2006; Nicholson et al., 2013). The 3′-CITE binds to eukaryotic translation initiation factor (eIF) 4F (Nicholson et al., 2010, 2013), which then gains access to the 5′-end of the genome through an LDRI (spanning ∼4.5 kb) that occurs between the 3′-CITE and the genomic 5′-UTR (**Figure 1B**; Fabian and White, 2004, 2006; Nicholson and White, 2008; Nicholson et al., 2013). When juxtaposed 5′-proximally, the 3′-CITE-bound eIF4F mediates recruitment of the small ribosomal subunit, which enters at the 5′-end of the genome, scans 5′-to-3′, and initiates translation at the start codon of p33 (Fabian and White, 2006; Nicholson et al., 2010). This process involves reiterative formation of the 5′-UTR–3′-CITE interaction to recruit the 43S subunit followed by disruption of the LDRI by scanning ribosomes (Fabian and White, 2006; Nicholson et al., 2010). The two smaller 3′-coterminal tombusvirus sg mRNAs also use this translational mechanism, because they too contain the 3′-CITE and have 5′-UTRs that can base pair with it (**Figure 1B**; Fabian and White, 2004; Nicholson and White, 2008). Thus, in addition to alleviating the need to either self-encode or hijack host capping and polyadenylating factors, the 3′-proximal placement of the CITE strategically allows for its use by both the genome and sg mRNAs. However, the presence of the complementary sg mRNA 5′-UTR sequences within the viral genome means that their access to the genomic 3′-CITE must somehow be prevented, because such interactions would competitively inhibit the 3′-CITE interacting with the 5′-UTR of the viral genome.

### Translational Readthrough

During infections, p92 RdRp accumulates to a level that is approximately 20-fold less than that for p33 auxiliary replication protein (Scholthof et al., 1995). These levels are consistent with p92 production via inefficient translational readthrough of the p33 stop codon. Investigation into the RNA elements required for tombusvirus translational readthrough revealed that two LDRIs are required for optimal readthrough production of the RdRp (Cimino et al., 2011). The first occurs between a bulge [proximal readthrough element (PRTE)] within an extended RNA stem–loop structure [readthrough stem loop (RTSL)] positioned just downstream of the p33 stop codon and the terminal loop of a hairpin [distal readthrough element (DRTE)] located in the 3′-UTR of the viral genome (**Figure 1B**). This PRTE–DRTE interaction spans ∼3.5 kb and is essential for efficient readthrough. Presumably, formation of this interaction directly or indirectly interferes with acquisition of translation release factors and/or facilitates recognition of near-cognate tRNAs that can pair with the p33 UAG stop codon. Interestingly, the 3′-proximal DRTE is integrated with a small RNA hairpin that is important for genomic minus-strand RNA synthesis (Fabian et al., 2003; Na and White, 2006). Notably, this integrated readthrough/replication element can form two mutually exclusive RNA hairpins that function to either (i) facilitate readthrough and repress minus-strand synthesis or (ii) inhibit readthrough and promote minus-strand synthesis (Cimino et al., 2011). Accordingly, the LDRI-based communication of the RTSL with the 3′-end of the genome also assists in coordinating these two processes, which proceed in opposite directions on the viral genome (Cimino et al., 2011).

Efficient readthrough also requires a second LDRI (Cimino et al., 2011). This interaction was first discovered as one that facilitates viral RNA replication and will be discussed further in the next section (Wu et al., 2009). This additional LDRI involves complementary sequences located near the 3′-UTR, termed downstream linker (DL), and one in the p92 coding region, termed upstream linker (UL) (**Figure 1C**). The UL–DL interaction is located between the PRTE–DRTE interaction; thus, it is hypothesized that the former assists in bringing the latter’s partner sequences into proximity, thereby promoting their pairing (Cimino et al., 2011). Consequently, two distinct LDRIs are necessary for productive translational readthrough, indicating that defined folding of a large section of the viral genome is required for this process.

### Genome Replication

*Tomato bushy stunt virus* genome replication takes place after the auxiliary replication proteins p33 and p92 RdRp are translated. The synthesis of progeny genomes requires the assembly of p33/p92 and host factors into a viral replicase complex (VRC) (Nagy, 2016). The VRC is responsible for copying the genome into a complementary minus-strand RNA that is then used as a template for the synthesis of multiple positive-sense progeny RNA genomes. Interestingly, two local RNA structures, separated by ∼3 kb, are required for VRC assembly (Monkewich et al., 2005; Pogany et al., 2005; Wu et al., 2009; Pathak et al., 2012). The first structure, termed RII, binds to p33/92 and is located in the coding region for p92 (**Figure 1C**; Monkewich et al., 2005; Pogany et al., 2005). The second structure, termed RIV, interacts with host factors eEF1A and eEF1Bγ (Li et al., 2009, 2010; Sasvari et al., 2011) and corresponds to the 3′-terminal portion of the viral genome. These two essential RNA structures, RII and RIV, are located ∼3 kb apart from one another, but are united by an LDRI formed between the aforementioned complementary UL and DL sequences (**Figure 1C**). Disruption of this interaction inhibits VRC formation and genome replication, confirming the requirement for RII and RIV to be juxtaposed for function (Wu et al., 2009; Pathak et al., 2012). Hence, the role of the UL–DL interaction in this viral process is to generate a bipartite RII–RIV RNA platform needed for VRC assembly (Pogany et al., 2005; Wu et al., 2009; Li et al., 2010; Sasvari et al., 2011; Pathak et al., 2012). Notably, because RII is absent in both of the sg mRNAs, VRC assembly is restricted to the genomic RNA.

### Subgenomic mRNA Transcription

Proteins encoded 3′-proximally in the TBSV genome (i.e., p41, p22, and p19) are translationally silent within that genomic context. Consequently, their expression requires transcription of two sg mRNAs during infections (White and Nagy, 2004). Tombusvirus sg mRNAs are synthesized through a mechanism involving premature termination (PT) of the p92 RdRp (White, 2002; Jiwan and White, 2011). In this process, the TBSV RdRp initiates minus-strand synthesis at the 3′-end of the viral genome. However, instead of copying the genome completely, it terminates prematurely at two defined internal sites (one for each sg mRNA) when it encounters RNA elements termed attenuation structures. The truncated minus strands generated contain promoters for plus-strand synthesis at their 3′-ends and are subsequently used as templates to transcribe sg mRNAs. The attenuation structures are RNA structures within the viral genome that act as physical barriers for the viral polymerase, causing it to prematurely terminate genomic minus-strand synthesis (Jiwan and White, 2011). In tombusviruses, attenuation structures are not represented by local structures and, instead, are formed by LDRIs. Formation of the sg mRNA1 attenuation structure of TBSV requires a base pairing interaction traversing the latter portion of the region coding for p92. The interaction, between activating sequence 1 (AS1) and receptor sequence 1 (RS1), spans ∼1 kb (**Figure 1D**; Choi and White, 2002; Lin et al., 2007). For sg mRNA2, the attenuation structure is formed through a comparable, but different, LDRI, involving AS2 and RS2 that are separated by ∼2 kb (Lin and White, 2004). Interestingly, sg mRNA2 also requires an additional LDRI located between the AS2–RS2 interaction, involving distal element (DE) and core element (CE) (**Figure 1D**; Zhang et al., 1999; Choi et al., 2001). Similar to the proposed role for the UL–DL interaction in translational readthrough, the DE–CE interaction is conjectured to help bring AS2 and RS2 into proximity, thereby facilitating their base pairing. Tombusviruses likely utilize transcriptional attenuation structures formed by LDRIs because the distal positioning of partner sequences provides superior control of their formation (i.e., via the kinetics of RNA folding or the involvement of protein factors). Additionally, such global folding could possibly facilitate cross-talk with tombusvirus LDRIs involved in other processes, leading to enhanced viral fitness during infections.

### Tombusvirus LDRIs: Six and Counting

Currently, six different LDRIs have been identified that perform distinct roles during tombusvirus infections. This complex and dynamic network of RNA–RNA interactions assists the virus by performing and coordinating critical viral processes, allowing each to occur at the appropriate time during infections, without interference between processes (**Figure 1A**). In the predicted LDRI cascade, the 5′-UTR–3′-CITE LDRI would form first to allow for translation initiation and synthesis of p33 (**Figure 1B**). Next, interacting PRTE–DRTE and UL–DL sequences would mediate readthrough production of the p92 polymerase (**Figure 1B**). This would be followed by formation of the UL–DL interaction that directs VRC assembly needed for RNA genome replication (**Figure 1C**). The AS–RS and auxiliary DE–CE interactions would then allow for transcription of the sg mRNAs (**Figure 1D**), and their ensuing translation would be facilitated by their cognate 5′-UTR–3′-CITE LDRIs (**Figure 1B**). Such structural choreography is envisioned to require a high level of genomic organization, and results from structural studies on the full-length TBSV RNA genome are consistent with this concept (Wu et al., 2013).

Why TBSV and other viruses have adopted this form of large-scale RNA communication for regulatory purposes is unclear. One can imagine that a physically compact RNA genome could provide a structural context favorable for sampling the utility of different LDRIs over time, with beneficial ones being maintained. The addition of an LDRI would add further complexity and possibly new pairing opportunities, thus the network could continue to grow, with new LDRIs being integrated with the existing system. Coordination and cross-talk between different LDRIs is likely integral to their function and this type of regulatory mechanism could offer certain advantages not available to other systems.

Tombusviruses appear to represent an excessive case with respect to LDRIs; however, these interactions are not limited to this genus, and many LDRIs have been reported in other members of *Tombusviridae*, as well as in other plus-strand RNA plant viruses. Below we provide a survey of the involvement of LDRIs in different processes in these other groups of viruses.

## LDRIs in Other Tombusvirids

The family *Tombusviridae* currently comprises 16 genera. Within this grouping, several species in different virus genera have been well characterized and these studies have identified LDRIs that operate in different viral processes. As LDRIs in many of these viruses share the same function, to avoid redundancy, below they are described collectively based on their type of activity. For a summary of the different functional LDRIs present in each of the viruses described, readers are directed to **Table 1**.

**Table 1:** Summary of documented LDRIs of different plant virus genera.

Virus	Translation initiation	Translational recoding	Genome replication	Subgenomic mRNA transcription	References
Tombusvirus: TBSV CIRV	+ +	+^a^	+ +	+	Zhang et al., 1999; Choi and White, 2002; Fabian and White, 2004; Lin and White, 2004; Nicholson and White, 2008; Wu et al., 2009; Cimino et al., 2011
Aureusvirus: CLSV	+		+	+	Xu and White, 2008; Xu and White, 2009; Lee and White, 2014
Pelarspovirus: PLPV	+			+	Blanco-Pérez et al., 2016; Blanco-Pérez and Hernández, 2016
Umbravirus: PEMV2	+	+^b^			Gao et al., 2012; Gao and Simon, 2016
Dianthovirus: RCNMV		+^b^		+	Sit et al., 1998; Tajima et al., 2011
Alphacarmovirus: SCV	+				Chattopadhyay et al., 2011
Betacarmovirus: TCV		+^a^			Cimino et al., 2011
Betanecrovirus: TNV-D		+^a^			Newburn et al., 2014
Luteovirus: BYDV	+	+^b^			Guo et al., 2001; Barry and Miller, 2002
Nepovirus: BRV	+				Karetnikov et al., 2006; Karetnikov and Lehto, 2007b
Potexvirus: PVX			+		Kim and Hemenway, 1999; Hu et al., 2007

*A plus sign indicates which virus utilizes an LDRI for a particular viral process. The superscript “a” and “b” correspond to translational readthrough and -1 frameshifting, respectively. CIRV, Carnation ringspot virus; CLSV, Cucumber leafspot virus.*

### Translation Initiation

**FIGURE 2:**
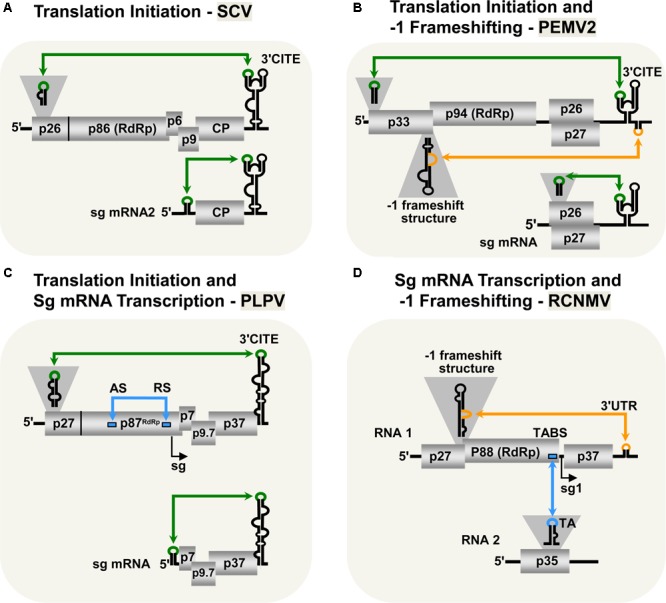
LDRIs in other tombusvirids. **(A)** LDRIs between the 3′-CITE and 5′ stem–loop structures (green) important for translation initiation from *Saguaro cactus virus* (SCV, genus *Alphacarmovirus*) genome and sg mRNA2. **(B)** LDRIs regulating translation initiation in *Pea enation mosaic virus-2* (PEMV2, genus *Umbravirus*) genomic and sg mRNAs (green), and –1 frameshifting in the genome (orange). **(C)** LDRIs involved in translation initiation (green) in *Pelargonium line pattern virus* (PLPV, genus *Pelarspovirus*) genomic and sg mRNAs along with an LDRI forming the attenuation structure for sg mRNA transcription (blue). **(D)** LDRI necessary for –1 frameshifting (orange) and a *trans*-interaction needed for sg mRNA transcription (blue) in *Red clover necrotic mosaic virus* (RCNMV, genus *Dianthovirus*).

Viruses in the family *Tombusviridae* lack both 5′-cap and 3′-poly(A) tail structures and instead possess different types of 3′-CITEs (Kneller et al., 2006; Miller and White, 2006; Nicholson and White, 2011; Simon and Miller, 2013; Truniger et al., 2017). Many of these viruses have also been shown to use LDRIs for communication between their 3′-CITEs and cognate 5′-UTRs. The genus *Aureusvirus*, which is most closely related to tombusviruses, possesses comparable proteins and coding organization (Rubino et al., 1995a,b; Miller et al., 1997; Martelli et al., 1998), and utilizes analogous LDRIs for protein translation (Xu and White, 2009). Similarly, LDRIs between the 5′-UTRs of genomic or sg mRNAs and their cognate 3′-CITEs are also required in other tombusvirids, such as *Saguaro cactus virus* (SCV, genus *Alphacarmovirus*) (Chattopadhyay et al., 2011), *Pea enation mosaic virus 2* (PEMV2, genus *Umbravirus*) (Gao et al., 2012; Gao and Simon, 2017), and *Pelargonium line pattern virus* (PLPV, genus *Pelarspovirus*) (**Figures 2A–C**; Blanco-Pérez et al., 2016). Although the 3′-CITEs of the abovementioned tombusvirids are distinct and interact with different translation initiation factors and/or ribosomal subunits (Gazo et al., 2004; Wang et al., 2009; Nicholson and White, 2011; Gao et al., 2012; Simon and Miller, 2013; Truniger et al., 2017) the function of their 5′–3′-LDRIs is thought to be the same: i.e., pseudo-circularization of the mRNA to deliver 3′-CITE-bound eIFs and/or ribosomes to the 5′-end of the mRNA to enhance translation initiation (Simon and Miller, 2013). This RNA-based pseudo-circularization may also provide some of the benefits proposed for protein-based pseudo-circularization of cellular mRNAs, such as providing a quality check for complete messages and facilitating the recycling of ribosomes.

3′-CITE-associated LDRIs appear to be common in tombusvirids (Simon and Miller, 2013) and conserved among all pelarspoviruses (Blanco-Pérez et al., 2016), some members of the alpha-, beta-, and gamma-carmoviruses (Chattopadhyay et al., 2011), as well as, in other genera of the family *Tombusviridae* (Fabian and White, 2004). However, for some tombusvirids, i.e., *Tobacco necrosis virus* (TNV-D, genus *Betanecrovirus*) and *Red clover necrotic mosaic virus* (RCNMV, genus *Dianthovirus*), tentatively identified translation-related 5′–3′-LDRIs were later proven to not be required for efficient translation of viral proteins (Sarawaneeyaruk et al., 2009; Chkuaseli et al., 2015). Accordingly, potential LDRIs need to be experimentally validated before function can be ascribed conclusively.

### Translational Readthrough and -1 Frameshifting

Most tombusvirids produce their RdRps through a translational readthrough mechanism, similar to that described for tombusviruses (Cimino et al., 2011). The genomes of TNV-D and *Turnip crinkle virus* (TCV, genus *Betacarmovirus*) both require LDRIs between an RNA structure proximal to the readthrough site and their 3′-UTR for optimal readthrough of their RdRps (Cimino et al., 2011; Newburn et al., 2014; Kuhlmann et al., 2016; Newburn and White, 2017). Comparable LDRIs are also predicted for other tombusvirids that use readthrough for RdRp production (e.g., *Aureusvirus, Panicovirus, Machlomovirus*, and *Avenavirus*); however, their activities have yet to be confirmed empirically (Cimino et al., 2011).

Only two genera in *Tombusviridae* use programed -1 ribosomal frameshifting for translation of their RdRps: *Umbravirus* (e.g., PEMV2) and *Dianthovirus* (e.g., RCNMV) (Tajima et al., 2011; Firth and Brierley, 2012; Gao and Simon, 2016). In both cases, frameshifting requires a conserved slippery heptanucleotide sequence at the frameshifting site and a 3′-adjacent RNA secondary structure (Delmer et al., 1993; Xiong et al., 1993; Kim and Lommel, 1998; Gao and Simon, 2016). However, for optimal frameshifting to occur, the shift site-adjacent RNA structures need to base pair with distal 3′-proximal sequences via LDRIs, in a manner similar to that described for readthrough (**Figures 2B,D**; Tajima et al., 2011; Gao and Simon, 2016). Such LDRIs may stabilize the shift site-adjacent RNA structure and assist back-stepping of ribosomes at the slippery heptanucleotide sequence (Barry and Miller, 2002; Tajima et al., 2011; Gao and Simon, 2016). This type of LDRI is also predicted to occur in six other members of the *Umbravirus* genus (Gao and Simon, 2016).

### Genome Replication

Like tombusviruses, other tombusvirids replicate their genomes using their encoded RdRps and auxiliary replication proteins. This process is regulated by a variety of *cis*-acting RNA elements positioned at different locations within the viral genome, and in some cases, like for TBSV, these RNA elements communicate through LDRIs (Wu et al., 2009). Indeed, aureusviruses contain close counterparts of the RII and RIV replication structures present in tombusviruses and, similarly, an LDRI is required to unite these structures for VRC assembly (Lee and White, 2014). Interestingly, replication-related RNA structures akin to both RII (Nicholson et al., 2012) and RIV (Na and White, 2006) have also been identified in alpha/betanecroviruses, alpha/beta/gammacarmoviruses, and pelarspoviruses; however, it has not yet been established that these RNA elements also require communication via LDRIs.

### Sg mRNA Transcription

Tombusvirids transcribe sg mRNAs during infections to allow for efficient translation of 3′-encoded genes (White, 2002; Jiwan and White, 2011). As for tombusviruses, the PT mechanism of sg mRNA transcription has been proposed for dianthoviruses (Sit et al., 1998), carmoviruses (Wu et al., 2010), aureusviruses (Xu and White, 2008, 2009), pelarspoviruses (Blanco-Pérez and Hernández, 2016), and betanecroviruses (Jiwan et al., 2011). Of these five genera, only aureusviruses (not shown) and pelarspoviruses (**Figure 2C**) were demonstrated to utilize LDRIs to form their transcription attenuation structures (Xu and White, 2008; Blanco-Pérez and Hernández, 2016). Interestingly, aureusviruses use an LDRI only for sg mRNA2 transcription, because the attenuation structure for sg mRNA1 is formed locally (Xu and White, 2008, 2009). The attenuation structures of betanecroviruses and carmoviruses are generated by local RNA secondary structures (Wu et al., 2010; Jiwan et al., 2011), while that for dianthoviruses forms, *in trans*, between its two genomic segments (**Figure 2D**; Sit et al., 1998). In the case of dianthoviruses, the *trans*-interaction that activates transcription of the capsid protein-encoding sg mRNA occurs when the levels of the genome segments are high, thereby appropriately inducing CP production and packaging late in the infection. Exactly how *cis*-acting LDRIs or local attenuation structures mediate temporal control of sg mRNA transcription remains an area of study that requires further investigation.

## LDRIs in Non-Tombusvirids

Long-distance RNA–RNA interactions are not limited to tombusvirids. Other plus-sense RNA plant viruses in the families *Luteoviridae, Secoviridae*, and *Alphaflexviridae* have been shown to regulate different viral processes through LDRIs. Below is a description of these LDRIs.

### Translation Initiation

**FIGURE 3:**
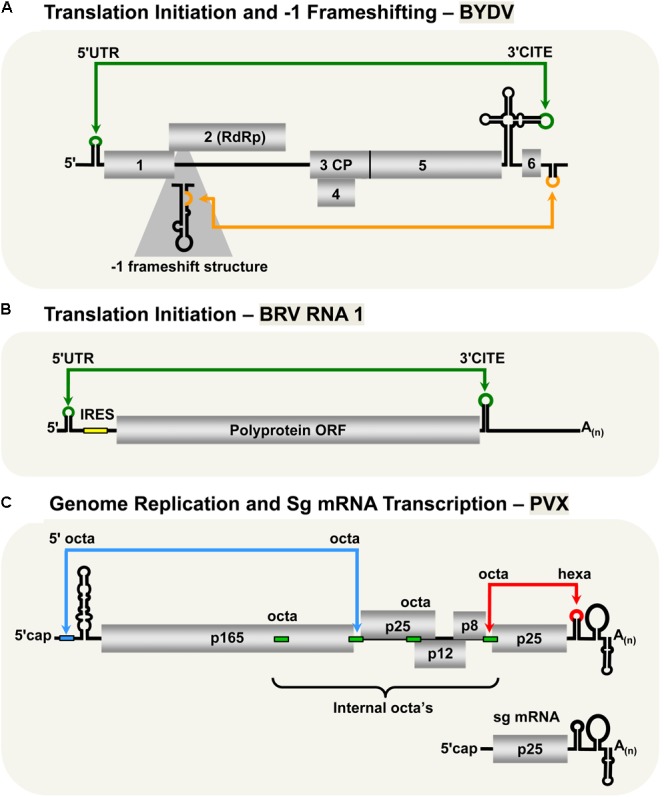
LDRIs in non-tombusvirids. **(A)** LDRIs that regulate translation initiation (green) and –1 frameshifting (orange) in *Barley yellow dwarf virus* (BYDV, genus *Luteovirus*, family *Luteoviridae*) genomic RNA. **(B)** The LDRI required for translation initiation (green) in *Blackcurrant reversion viru*s (BRV, genus *Nepovirus*, family *Secoviridae*) RNA1. The small yellow box represents an internal ribosomal entry site (IRES) that also facilitates translation initiation along with the 3′-CITE and the 3′-poly(A) tail. **(C)** LDRIs between the 5′-octa and an internal octa (green rectangles) in *Potato virus X* (genus *Potexvirus*, family *Alphaflexiviridae*) needed for plus-strand accumulation of genomic and sg mRNAs (blue) and between hexa and an internal octa required for synthesis of minus-strand RNAs (red). Note that only two of multiple different possible interactions are shown.

The *Luteovirus, Barley yellow dwarf virus* (BYDV, family *Luteoviridae*) utilizes similar cap-independent translation initiation mechanism as members of the family *Tombusviridae* described in previous sections. Indeed, due to this and other likenesses, it has been suggested that BYDV and other luteoviruses should be reclassified as tombusvirids (Miller et al., 2002). BYDV possesses a 3′-CITE that binds eIF4F (Guo et al., 2000; Treder et al., 2008) and base pairs with a 5′-complementary sequence via a kissing-loop LDRI spanning ∼4.5 kb (**Figure 3A**; Guo et al., 2001). Importantly, this was the first experimental confirmation of the involvement of a LDRI in cap-independent translation (Guo et al., 2001). Also, it was shown that moving the 3′-CITE to the 5′-UTR of the viral genome obviated the need for the 5′–3′-LDRI, thus confirming the role of the LDRIs in repositioning the 3′-CITE-bound eIFs to the 5′-end of the genome (Guo et al., 2000). Like for tombusviruses, the 3′-CITE-mediated translation initiation likely involves repetitive formation and disruption of the kissing-loop LDRI during the translation process (Rakotondrafara et al., 2006). This type of LDRI is also predicted to function for BYDV sg mRNA1 translation, as well as in *Soybean dwarf virus* (genus, *Luteovirus*) (Guo et al., 2001); however, these proposals await experimental validation.

Unlike all the plant viruses discussed in the previous sections, the two RNA genomic segments of *Blackcurrant reversion virus* (BRV, genus *Nepovirus*, family *Secoviridae*) have a 3′-poly(A) tail, but lack a 5′-cap structure (**Figure 3B**; Latvala-Kilby and Lehto, 1999; Pacot-Hiriart et al., 2001). The 3′-poly(A) tail was shown to be important for optimal protein translation from BRV RNA1- and RNA2-based reporter constructs (Karetnikov et al., 2006; Karetnikov and Lehto, 2007b). Additionally, the 5′-UTRs of the genomic RNAs were shown to harbor internal ribosomal entry sites (IRESes), also important for efficient translation (Karetnikov and Lehto, 2007a,b). Interestingly, both of the BRV genomic RNAs also contain 3′-CITEs in their 3′-UTRs that communicate with their 5′-UTRs through a kissing-loop LDRI (Karetnikov et al., 2006; Karetnikov and Lehto, 2007b). It was also proposed that these LDRIs could enhance genome translation by facilitating recycling of the ribosomal subunits back to the IRESes located in their 5′-UTRs (Karetnikov and Lehto, 2007b).

### Translational Readthrough and -1 Frameshifting

*Barley yellow dwarf virus* expresses its RdRp through -1 frameshifting, similar to RCNMV and PEMV2 (Brault and Miller, 1992; Paul et al., 2001). Optimal frameshifting in BYDV requires a LDRI between the 3′-UTR and a stem–loop structure near the frameshifting site across ∼4 kb (**Figure 3A**; Barry and Miller, 2002). The proposed role of this LDRI is similar to that for the RCNMV and PEMV2 -1 frameshift-promoting LDRIs; that is, stabilizing the shift site-proximal structure, causing stalling of the ribosomes thereby stimulating the -1 frameshift (Barry and Miller, 2002).

In addition to using -1 frameshifting as a translation mechanism, BYDV translates a C-terminally extended coat protein through a readthrough mechanism from its sg mRNA1 (Dinesh-Kumar et al., 1992). It was shown that the coat protein stop codon readthrough requires sequences close to the readthrough site, as well as sequences ∼0.7 kb downstream. It was proposed that this DRTE communicates with the readthrough-proximal element via a LDRI; however, this has not yet been verified experimentally (Brown et al., 1996).

### Genome Replication and sg mRNA Transcription

The type species of the genus *Potexvirus* (family *Alphaflexiviridae*), *Potato virus X* (PVX), has an interesting genome replication and sg mRNA transcription mechanism that involves multiple LDRIs (Kim and Hemenway, 1999; Batten et al., 2003; Hu et al., 2007). The RNA genome of PVX contains several RNA sequences involved in these LDRIs: an octanucleotide (octa) sequence in its 5′-UTR, four internal octa sequences, and a hexanucleotide (hexa) sequence in its 3′-UTR (**Figure 3C**; Kim and Hemenway, 1996, 1997, 1999; Pillai-Nair et al., 2003; Hu et al., 2007). Notably, both the octa sequence in the 5′-UTR and the hexa sequence in the 3′-UTR can base pair with any of the four internal octa sequences via LDRIs. Interaction between the 3′-UTR hexa sequence and one of the four internal octa sequences is critical for synthesis of the minus-sense genome (Hu et al., 2007), whereas genomic and sg plus-strand RNA accumulation requires base pairing of the 5′-UTR octa with one of the internal octa sequences (Kim and Hemenway, 1999). It was proposed that the large number of possible combinations of interactions between the internal and terminal sequences could provide a means to control the levels of plus and minus strand RNAs, as well as coordinate the timing of genome replication and sg mRNA transcription (Hu et al., 2007).

## Conclusion and Perspective

This survey of LDRIs in plus-strand RNA plant viruses illustrates both their prevalence in different virus classes and their diversity of function (**Table 1**). These distance-spanning structures represent an additional layer of RNA-based control that provides novel regulatory mechanisms not possible with local RNA structures. Additionally, to ensure optimal function of all processes, LDRIs are highly integrated and coordinated with other RNA elements, as well as with the multiple functions of the viral genome. Accordingly, understanding this complex context in which LDRIs operate will require a holistic perspective when investigating viral genome structure and function.

Significant progress has been made in identifying and understanding the functions and mechanisms of LDRIs, however, much remains unknown (Miller and White, 2006; Nicholson and White, 2014). For many of the LDRIs already discovered, general features of how they function have been uncovered, however, other important aspects of their activities require further analysis. For instance, the factors that determine how and when LDRIs form remain largely unknown. In most cases, thermodynamic stability is likely to be important; however, it is quite probable that some LDRIs also require viral and/or host proteins to mediate their formation. Conversely, LDRIs also need to be inactive at certain times, leaving open the question of how they are disrupted or prevented from forming.

The dynamic nature of these RNA elements, combined with their distance-spanning nature, makes them a challenge to study. Nonetheless, with the advent of high-throughput chemical probing techniques such as selective 2’-hydroxyl acylation analyzed by primer extension (SHAPE) it is now possible to gain insights into complete viral genome higher-order structures (Low and Weeks, 2010). Such global structural contexts have been integral to understanding how LDRIs are accommodated within viral genomes (Wu et al., 2013). Although such studies generate only a “snapshot” of a predicted dominant structure in a genomic population, they do provide a starting point for building a genomic model that includes LDRIs.

The discovery of new LDRIs also presents a challenge (Lim and Brown, 2018). Many of the initial discoveries were made serendipitously based on the codependence of distant regions for a particular function. These examples then provided templates for others to carry out more systematic searches for corresponding interactions in other viruses. *De novo* identification is possible using computational approaches, however, some LDRIs may be missed, and only a small subset is likely to be functional. Regardless, performing viral genome folding using RNA secondary structure predicting programs such as mFOLD can be informative regarding potential LDRIs (Zuker, 2003). Additionally, programs such as intraRNA and LRIscan designed specifically for identifying interactions based on the extent of pairing and/or the presence of covarying base pairs can also be useful (Wright et al., 2014; Fricke and Marz, 2016). Any putative functional LDRIs identified can then be assessed functionally using compensatory mutational analyses, and contacts can be verified by solution structure probing.

A final interesting aspect, for which essentially nothing is known, is how LDRIs initially arise and how they evolve over time. With the popularity of high-throughput sequencing, which allows for facile discovery of new plant viruses, novel viral sequences are being published at an ever-growing rate (Roossinck, 2017). Among these will be both close and more distant relatives of known viruses that possess LDRIs. Thus, by examining this pool it may be possible to identify the emergence and/or transitions of different LDRIs. Such analyses, along with the discovery of new LDRIs and further studies of existing LDRIs, will undoubtedly expand the field and answer many outstanding questions in this fascinating area of research.

## Author Contributions

Both authors have made a substantial direct and intellectual contribution to the work and approved it for publication.

## Conflict of Interest Statement

The authors declare that the research was conducted in the absence of any commercial or financial relationships that could be construed as a potential conflict of interest.
